# Enzymatic Saccharification of Lignocellulosic Residues by Cellulases Obtained from Solid State Fermentation Using *Trichoderma viride*

**DOI:** 10.1155/2015/342716

**Published:** 2015-06-02

**Authors:** Tanara Sartori, Heloisa Tibolla, Elenizi Prigol, Luciane Maria Colla, Jorge Alberto Vieira Costa, Telma Elita Bertolin

**Affiliations:** ^1^Laboratory of Fermentations, Course of Food Engineering, School of Engineering and Architecture, University of Passo Fundo, Campus I, 99052900 Passo Fundo, RS, Brazil; ^2^Department of Food Engineering, School of Food Engineering, University of Campinas, Campinas, SP, Brazil; ^3^Laboratory of Biochemical Engineering, School of Chemistry and Food, Federal University Foundation of Rio Grande, Rio Grande, RS, Brazil

## Abstract

The aim of this study was to verify the viability of lignocellulosic substrates to obtain renewable energy source, through characterization of the cellulolytic complex, which was obtained by solid state fermentation using *Trichoderma viride*. Enzymatic activity of the cellulosic complex was measured during saccharification of substrates filter paper, eucalyptus sawdust, and corncob, and compared with the activity of commercial cellulase. The characterization of the enzymes was performed by a 2^2^ Full Factorial Design, where the pH and temperature were the variables of study. Enzymatic saccharification of different substrates appearedviable until 12 to be viable until 12 h; after this period the activity decreased for both enzymatic forms (cellulolytic complex and commercial cellulase). The enzymatic activity of the commercial cellulase was favored with the use of corncob as substrate, while the cellulolytic complex does not show any difference in its specificity by the substrates studied. The largest activities of both enzymes were obtained in the temperature and pH range between 40°C and 50°C and 4.8 and 5.2, respectively. The cellulolytic complex obtained appeared to be viable for the saccharification of lignocellulosic residues compared with the commercial cellulase.

## 1. Introduction

The growing demand for energy for transportation and industrial processes stimulates the search for new energetic renewable matrixes to replace fossil fuels that have limited reserves, turning feasible the use of agroindustrial residues that besides being abundant reduce the environmental impact [1, 2].

The lignocellulosic biomass is the largest source of carbohydrate, since it is the main plant cellular wall. It consists of lignins chains, cellulose, and hemicellulose, which are intertwined and chemically linked by noncovalent forces and by covalent crossed connections, becoming a substrate of difficult hydrolysis [3, 4].

The cellulolytic enzymes are synthesized by microorganisms like bacteria and fungi.* Cellulomonas fimi*,* Clostridium thermocellum* [5], and* Bacillus subtilis* [6] stand out among the cellulose-producing bacteria. The most studied species of filamentous fungi are* Trichoderma viride* [7],* Aspergillus niger* [8],* Penicillium funiculosum* [9], and* Rhizopus oligosporus* [10]. These enzymes act in synergism breaking glucosidic bonds type *β*-1.4 from the cellulose chain, resulting in the release of oligosaccharides, cellobiose, and glucose [11].

The greatest difficulty for the use of the lignocellulosic residues is represented by the physical barrier formed by the lignin, which prevents the use of the native cellulose; thus the enzymes cannot penetrate this barrier easily. The separation of the lignin may be achieved through physical, chemical, or biological treatments or their combination. The chemical treatment is usually used through acid or alkaline hydrolysis [11].

The most used techniques in the cellulose biomass hydrolysis are the chemical and enzymatic methods. The chemical hydrolysis presents advantages because of its high rate and unnecessary pretreatment, but the enzymatic hydrolysis is superior to it, in several aspects, as, for instance, before the possibility to be performed at low temperatures (45°C–50°C) and atmospheric pressure, there is no subproducts formation, increasing the yield of fermentable sugar production. The enzymatic reactions may occur under mild conditions of pH (4.8) not causing corrosion problems in equipment. Thus, to reach high conversion of cellulose it is necessary to have high concentrations of enzymes, increasing the cost of production. Therefore, the study of microorganisms, which produce high productivity cellulases, is very important, as well as the development of economic production techniques [8].

Fermentation in solid state consists in the process of microbial growing in solid substrate, with enough moisture to guarantee the cells growing and metabolism, and does not exceed the maximum retention capacity of water of the solid matrix, that is, exempt of free water [12]. The materials used in the fermentation are resulting from raw materials, products, and/or agroindustrial residues, where these later show low or none commercial value [13].

The filamentous fungi present better capacity of growing under conditions of low levels of water.* Trichoderma *sp. is an important microorganism in the production of cellulases, demonstrating capacity to produce the enzyme from several substrates, like corncob [14, 15], rice straw [16], wheat straw [17], and sugarcane straw [18].

According to Leu and Zhu [19], the efficiency of the enzymatic saccharification depends on factors such as the type of pretreatment of the substrate and the catalytic action of the enzymes (inhibiting effect by the final product formed, deactivation or denaturation due to reaction time, temperature, stirring and pH, synergic actuation of cellulolytic complex enzymes, and enzymes and substrate concentrations). The control of these parameters, aiming at great conditions for the enzymatic hydrolysis, is important so that greater reaction yield is obtained.

In this context, the objective was to study the saccharification of lignocellulosic residues by the cellulases obtained by fermentation in solid state using* Trichoderma viride*, evaluating the enzymatic behavior during the process of saccharification of different substrates (filter paper, eucalyptus sawdust, and corncob) through a 2^2^ Full Factorial Design with three center points.

## 2. Material and Methods

### 2.1. Microorganism, Maintenance, and Inoculum Preparation

The microorganism used was* Trichoderma viride*, which was obtained from the Tropical Foundation of Researches and Technology André Tosello, Campinas, SP. It was kept in agar filter paper, containing (g·L^−1^) the following: KH_2_PO_4_, 1.0; (NH_4_)_2_SO_4_, 0.5; KCl, 0.5; MgSO_4_·7H_2_O, 0.2; CaCl_2_, 0.1; yeast extract, 0.5; filter paper Whatman number 1, 10.0; agar, 20.0. The maintenance of the cultures was performed in test tubes, which were kept under refrigeration at 4°C.

The inoculum was prepared in Erlenmeyer flasks of 1000 mL with 50 mL of medium agar filter paper, to which 1 mL of spores suspension was added, resulting from the growth in test tubes. The Erlenmeyer flasks were kept in an oven at 30°C for 7 d, for later suspension with the addition of Tween 80 0.1% sterilized, which were filtered in cotton, for later use as inoculum.

### 2.2. Delignification of the Substrate

The corncob was the substrate used in the fermentation processes, which was delignified according to the method adapted from Sukumaran et al. [8]. The treatment was performed from a substrate concentration (lignified raw material) of 10% (w/v) with alkaline solution (NaOH 0.25 mol·L^−1^). This mixture was placed in stainless steel flasks covered with aluminum foils for later autoclaving, remaining at 121°C for 1 h at 110 kPa. After cooling, the substrate was neutralized with H_2_SO_4_ 1 mol·L^−1^ in the proportion of 0.125 mL_acid_/mL_base_ until the pH is close to neutralization. The mixture was washed in flowing water in order to remove the excess of reagents by using sieves with opening of 40 mesh so that there is no loss of raw material.

The delignified substrate was submitted to drying in an oven at 35°C during 24 h for total removal of moisture, obtaining a 50% delignified substrate.

### 2.3. Medium Culture and Fermentation to Obtain the Cellulolytic Complex

The corncob was used as the source of carbon, which was passed through sieve whose opening was 1.18 mm (14 mesh). To this substrate was added 30% (v/w) of macro- and micronutrients solution, adapted from the method described by Aguiar et al. [20], containing (g·L^−1^) KH_2_PO_4_, 2.0; (NH_4_)_2_SO_4_, 1.4; CO(NH_2_)_2_, 0.3; MgSO_4_·7H_2_O, 0.3; CaCl_2_, 0.3; FeSO_4_·7H_2_O, 0.005; MnSO_4_, 0.00156; ZnSO_4_·7H_2_O, 0.0014; CoCl_2_·6H_2_O, 0.0020. The substrate moisture was adjusted in 63% with distilled water and the pH of the medium was adjusted in 4.8 with acid solution (H_2_SO_4_ 0.5 mol·L^−1^).

Solid state fermentation for the production of cellulolytic enzymes (cellulolytic complex) was carried out in Erlenmeyer flasks of 250 mL with 10 g of medium culture, 0.5 g filter paper, and 1.0 mL of spores suspension (inoculum), containing 10^9^ spores·mL^−1^. The concentration of spores in suspension was estimated by counting in microscope, using a Neubauer chamber. The experiments were incubated at 30°C for 192 h.

### 2.4. Saccharification of Different Substrates by Using the Cellulolytic Complex

The lignocellulosic substrates corncob (C), eucalyptus sawdust (ES), or filter paper (FP) delignified were used in the saccharification assays. The enzymes of the cellulolytic complex (CC) were obtained from the bran of solid state fermentation with* Trichoderma viride*. The commercial cellulase (CE) was used to compare yield of saccharification.

The saccharification of the substrates was performed by using a 2^2^ Full Factorial Design (FFD) with three central points. The studied variables were temperature and pH, according to the matrix of experiments showed in Table 1.

The experiments were made in Erlenmeyer flasks of 300 mL, containing 5 g of substrate and 75 mL of citrate buffer 0.05 mol·L^−1^, with pH variable according to the experimental planning. The mixture was submitted to a thermostatic bath (temperature variable according to the factorial design) for 10 min for medium adaptation, after 5 g of fermented bran containing the cellulolytic complex or 5 mL of commercial enzyme with dilution of 1 : 100 (v/v) was added. The saccharification was made during 24 h, without agitation. The experiments were performed in triplicate. The control experiments were performed by using citrate buffer to replace the source of enzymes.

The enzymatic activity of the cellulolytic complex and of the commercial enzyme was evaluated according to the filter paper assay (FPU) adapted from Ghose [21]. Enzyme cellulase is defined as a cellulolytic complex, which is formed by three different enzymes (endoglucanases, exoglucanases, and *β*-glicosidases). These enzymes act in synergism, in a cooperative association, producing substrates to one another [22, 23].

The efficiency of saccharification was evaluated through the reducing sugars content after filtration, using the 3.5-dinitrosalicylic acid (DNS) method by spectrophotometer at 546 nm, using glucose as standard [24].

The results were showed in units, where one enzymatic unit (U) is defined as a quantity of enzyme which is able to release 1 *μ*mol of reducing sugar per hour under the conditions of the experiment. Equation (1) was used for the calculation of the enzymatic activity:

(1) $$\begin{array}{c} \text{EA}=\text{RS}\cdot \frac{v_{e}}{E}\cdot \frac{1}{0.18\cdot t}, \end{array}$$

where EA is enzymatic activity (U/g or U/mL); RS is concentration of reducing sugars (mg/mL); *v*_*e*_ is volume of extract; *E* is volume of commercial cellulase (mL) or fermented bran mass (g); *t* is time of the reaction (*h*); 0.18 mg/*μ*mol of glucose is released.

### 2.5. Statistical Analysis

The results of the enzymatic activity obtained in the planning were analyzed through analysis of variance (ANOVA), with the estimated effects and regression coefficients being obtained.

## 3. Results and Discussion

### 3.1. Formation of Reducing Sugars and Enzymatic Activity during Saccharification of the Substrates

**Figure 1:**
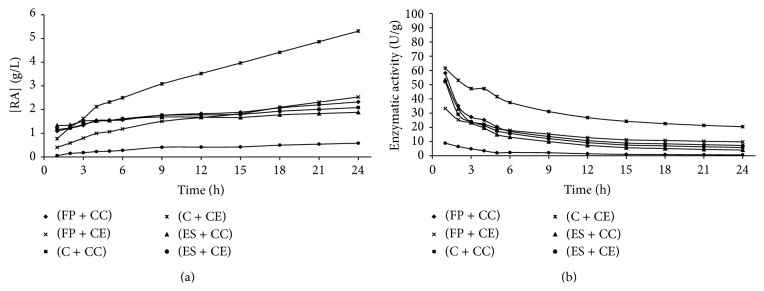
Concentration of reducing sugars formed (a) and enzymatic activity (b) during the time of saccharification, under experimental conditions of the temperature 40°C and pH of 4.4 (Experiment 1), where FP is filter paper, C is corncob, ES is eucalyptus sawdust, CC is cellulolytic complex, and CE is commercial enzyme.

**Figure 2:**
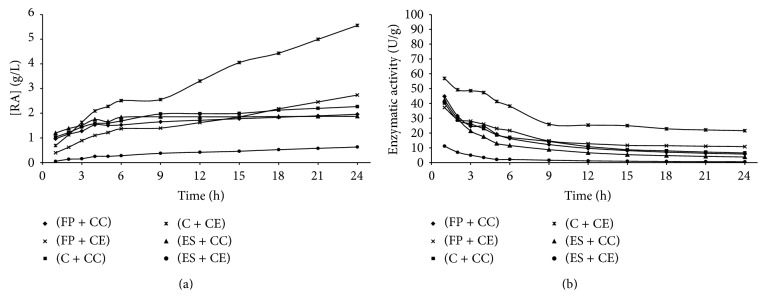
Concentration of reducing sugars formed (a) and enzymatic activity (b) during the time of saccharification, under experimental conditions of the temperature 40°C and pH of 5.2 (Experiment 2), where FP is filter paper, C is corncob, ES is eucalyptus sawdust, CC is cellulolytic complex, and CE is commercial enzyme.

**Figure 3:**
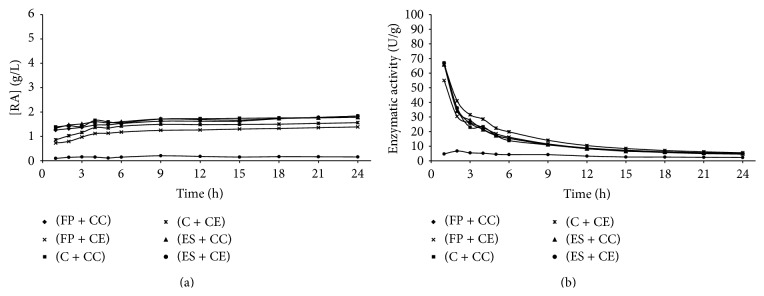
Concentration of reducing sugars formed (a) and enzymatic activity (b) during the time of saccharification, under experimental conditions of the temperature 60°C and pH of 4.4 (Experiment 3), where FP is filter paper, C is corncob, ES is eucalyptus sawdust, CC is cellulolytic complex, and CE is commercial enzyme.

**Figure 4:**
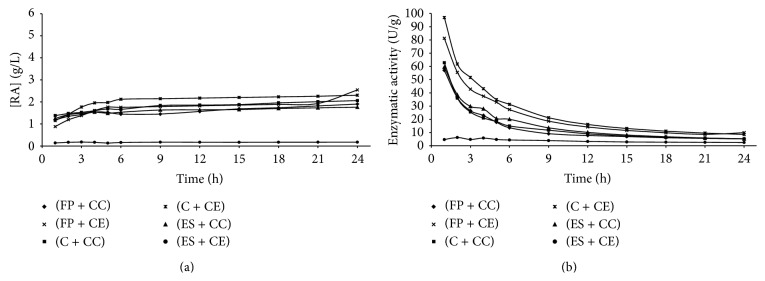
Concentration of reducing sugars formed (a) and enzymatic activity (b) during the time of saccharification, under experimental conditions of the temperature 60°C and pH of 5.2 (Experiment 4), where FP is filter paper, C is corncob, ES is eucalyptus sawdust, CC is cellulolytic complex, and CE is commercial enzyme.

**Figure 5:**
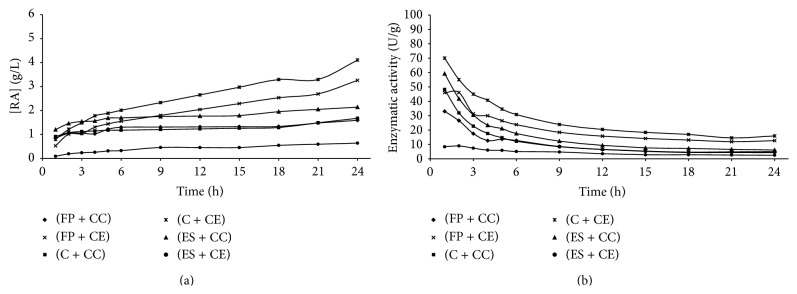
Concentration of reducing sugars formed (a) and enzymatic activity (b) during the time of saccharification, under experimental conditions of the temperature 50°C and pH of 4.8 (Experiment 5), where FP is filter paper, C is corncob, ES is eucalyptus sawdust, CC is cellulolytic complex, and CE is commercial enzyme.

Figures 1 to 5 present the reducing sugars concentrations formed and the activities of total cellulase during saccharification of the substrates filter paper (FP), corncob (C), and eucalyptus sawdust (ES) by using the cellulolytic complex (CC) obtained by fermentation in solid state and the commercial enzyme (CE).

The cellulolytic complex behavior was similar in all substrates studied during the saccharification period. This behavior was not observed for the commercial cellulase, which presented low conversion values when eucalyptus sawdust was used. Therefore, it was verified that the commercial enzyme has different degrees of specificity among substrates, while the cellulolytic complex did not present difference of specificity among the substrates, since no considerable differences in the values of enzymatic activity among the available substrates were presented (Figures 1(b)–4(b)). To the commercial enzyme, the corncob was the substrate which released greater quantities of reducing sugars, under experimental conditions of the temperature 40°C and pH of 4.4 and 5.2.

It was verified that the greatest enzymatic activities were obtained in initial times of saccharification, regardless of the source of enzyme used (cellulolytic complex or commercial cellulase). A high decrease in enzymatic activity was observed until 12 h of reaction, with later stabilization (Figures 1 to 5). For this reason, until 12 h of saccharification, the reducing sugars formation showed gradual increase, remaining stable after this period. This may be explained by the thermal denaturation of the enzymes and also by the influence of substrate concentration on the enzymatic activity, which according to Michaelis and Mentem predicts that the reaction rates increase because of the substrate concentration until a limit from which it passes to be constant [25, 26]. These results demonstrate that it is not feasible to continue the process of saccharification after 12 h of reaction, because of the reduction of the productivity in this period. Table 1 shows the results of the enzymatic activity in 12 h, obtained in saccharification of the filter paper, eucalyptus sawdust, and corncob, by using the cellulolytic complex obtained via SSF.

The maximum enzymatic activity of the cellulolytic complex on filter paper was shown in Experiment 1 (10.634 U/g), with temperature of 40°C and pH 4.4; thus it did not show significant difference (*P* > 0.05) relative to Experiment 2 (9.629 U/g), and this demonstrates that at the lower level of temperature (40°C) the effect of the pH variation was not significant. The same can be observed in Experiments 3 and 4 (60°C), in which the values of enzymatic activity do not show significant difference (*P* > 0.05) with variation of pH, with values of 8.421 U/g and 7.762 U/g, respectively. The results of the enzymatic activity obtained in the central points, performed under conditions mentioned in the literature as great conditions for cellulase action, do not appear to be the best actuation conditions of the cellulolytic complex on the filter paper. So, it can be observed that in one substrate of simple degradation (pure cellulose) the reaction is favored in the lower level of temperature (40°C).

The cellulolytic complex action on eucalyptus sawdust showed maximum enzymatic activity in the lower level of temperature (40°C) and higher level of pH (5.2), with value of 10.146 U/g. These conditions show no significant difference (*P* > 0.05) of the experiments of central points (50°C and pH 4.8).

The saccharification of the corncob substrate by the cellulolytic complex was favored under temperature of 40°C and pH 5.2 and this condition showed significant difference (*P* < 0.05) from the others. According to Marangoni [27], the enzymes present optimal ranges in determined temperature and pH conditions; thus they are characterized by their high specificity. The effect of the pH on the enzymatic activity is due to variations in the ionization state of the components of the system with the pH variation. As enzymes are proteins, they contain many ionizable groups; hence, the catalytic activity is restrict to a small level of pH.

Annamalai et al. [28] in order to study enzymatic saccharification pretreated rice straw by cellulase produced* Bacillus carboniphilus* CAS 3 utilizing lignocellulosic wastes found that the optimum temperature, pH, and NaCl for enzyme activity were determined as 50°C, 9, and 30% and more than 70% of its original activity was retained even at 80°C, 12, and 35%, respectively. In view of that, the authors suggest that higher temperature, pH, and halo stability of the purified cellulase could be useful for harsh industrial and various biotechnological applications.

Corncob and eucalyptus sawdust substrates show the lowest enzymatic activities in the experiments corresponding to temperature of 60°C. It is verified that the cellulolytic complex obtained a similar behavior in all substrates used, since this enzymatic form has greater performance in temperature between 40°C and 50°C and pH between 4.8 and 5.2. Gokhale et al. [29] observed that the enzymatic activity of cellulases increases gradually until temperature of 50°C and it reduces drastically in the temperature of 60°C, since there is loss of activity in higher temperatures because of the instability of the enzyme molecule. They also verified that the activity reduces when the pH is over 5.1, since the ionizable groups present in the structure of the enzymes make part of the catalytic site. By pH variations of medium, changes occur in its ionic form, resulting in change in the enzymes activity because of the reduction of its specificity.

By comparing maximum enzymatic activities obtained in the experiments of the FFD with the three substrates studied and by using, as source of enzymes, the cellulolytic complex obtained via SSF, it was verified that there was no significant difference (*P* > 0.05) between the averages of the enzymatic activity through the Tukey test at 5% of significance. This demonstrates that the delignification of the eucalyptus sawdust and corncob was efficient, since these substrates are complex, containing high amount of lignin in their original composition (25%), because the enzyme performed in a similar way to the substrate filter paper, presenting a simple composition (pure cellulose) in it.

Table 2 shows the results of the enzymatic activity of the commercial cellulase in the saccharification of the substrates filter paper, eucalyptus sawdust, and corncob.

Maximum enzymatic activities of the commercial cellulase on filter paper were obtained in the experiments of the central points (5, 6, and 7) with maximum value of 15.771 U/mL, at 50°C and pH 4.8, and there was no significant difference between them and Experiment 2 (40°C and pH 5.2), with value of 13.563 U/mL, as it can be observed in Table 2. In the saccharification of the eucalyptus sawdust, the best enzymatic activity was obtained in Experiment 5 (central point), with value of 3.692 U/mL, presenting significant difference from the other experiments (*P* < 0.05).

The commercial cellulase showed greater enzymatic activity in Experiment 1 of 26.787 U/mL using corncob as substrate; however, it did not present significant difference (*P* > 0.05) from Experiment 2, demonstrating that the pH did not influence the activity of this enzyme in intervals tested and at 40°C. The commercial cellulase activity decreases for all substrates with the transition from level −1 (40°C) to +1 (60°C). According to Marangoni [27], the temperature influence on the enzyme activity is represented in terms of activity or rate of reaction. With the increase of temperature an increase of the enzyme activity is observed until a point where the high temperature causes a thermal denaturation and the loss of the enzyme biological activity happens.

Comparing the averages of maximum enzymatic activities obtained in experiments of the FFD with three substrates studied and using the commercial cellulase, through the Tukey test at 5% of significance (15.771 U/mL for the filter paper, 3.692 U/mL for eucalyptus sawdust, and 26.787 U/mL for corncob), it was verified that there was significant difference (*P* < 0.05) between the averages obtained.

The commercial cellulase showed greater specificity with relation to the corncob and then with relation to the filter paper, and it was less specific for eucalyptus sawdust, showing maximum enzymatic activity value for this substrate, approximately 90% lower than the more specific substrate.

According to Sun and Cheng [30], the action of the enzymes may be affected because of the heterogeneity of many lignocellulolytic substrates, commonly used in biochemical processes, since they present varied amounts of cellulose, hemicellulose, and lignin in their biomass composition.

When comparing both forms of cellulase enzyme used, it is observed that the cellulolytic complex from* Trichoderma viride* showed lower enzymatic activities than the commercial cellulase in saccharification of the filter paper (approximately 30% lower) and the corncob (approximately 50% lower) and higher activities for the eucalyptus sawdust substrate, which could be attributed to better endoglucanases and cellobiohydrolases produced by* T*.* viride* [31]. The cellulase complex secreted by filamentous fungi consists of three major enzyme components, an endo-1,4-*β*-glucanase (EC 3.2.1.4), a 1,4-*β*-D-cellobiohydrolase (EC 3.2.1.91), and a 1,4-*β*-glucosidase (EC 3.2.1.21), which act synergistically. Although* T. reesei* produces cellobiohydrolases and endoglucanases in high quantities, it is deficient in *β*-glucosidase [32]. The results obtained were satisfactory, because the cellulolytic complex used was the gross product obtained by fermentation in solid state (fermented bran), since it was not submitted to the purification or genetic modification processes of microorganisms.

According to Le Ngoc Huyen et al. [33] the sustainability of ethanol production from lignocellulosic biomass would imply reduction in the consumption of chemicals and/or energetic means but also valorization of the lignocellulosic by-product remaining from enzymatic saccharification. To use lignocellulose as a feedstock, however, pretreatment is necessary to achieve industrially relevant rates of enzymatic saccharification. The pretreatment and enzymatic saccharification processes are costly and the outcomes are not easily predictable. A fundamental understanding of how pretreatment impacts enzyme-substrate interactions, for example, cellulase access to cellulose, can improve the predictability of process outcomes towards overall cost reductions [34].

Table 3 shows the analysis of variance and the effects estimated from variables pH and temperature on the enzymatic activity of the cellulolytic complex and the commercial cellulase, using different substrates in saccharification.

The analysis of variance (Table 3) shows that the enzymatic activity of the cellulolytic complex did not have significant influence (*P* > 0.05) from variables pH and temperature in saccharification of substrates filter paper and eucalyptus sawdust. But as for the corncob, variables temperature and pH were significant (*P* < 0.05). The temperature showed linear effect on the enzymatic activity (−1.361 U/g); that is, by varying the temperature of level +1 (60°C) to −1 (40°C), the enzymatic activity of the cellulolytic complex is favored, while variable pH showed positive linear effect (1.153 U/g); therefore, varying the pH from lower level (4.4) to higher level (5.2), an increase in the enzymatic activity occurs.

The enzymatic activity of the commercial cellulase on substrates filter paper and eucalyptus sawdust was not significantly influenced by variables pH and temperature (*P* > 0.05). With relation to substrate corncob, variable temperature influenced significantly, considering a confidence level of 90% (*P* = 0.06) in the enzymatic activity of the commercial cellulase, presenting a negative linear effect (−12.787 U/mL); that is, by transition from the temperature of the lower level, an increase in the catalytic reaction rate occurred.

## 4. Conclusions

The enzymes of the cellulolytic complex and the commercial cellulase present better activity and stability between 40°C and 50°C, and the enzymatic activity is not significantly influenced at pH (4.4–5.2). Considering the effect of the temperature on the enzymatic activity, the enzymes of the cellulolytic complex are less affected by the increase of temperature than the commercial cellulase enzyme. The cellulolytic complex shows high specificity for pure substrate as well as for complex substrates such as delignified corncob and eucalyptus. On the other hand, the commercial cellulase is more specific to corncob, followed by the pure substrate (filter paper). The results obtained demonstrate that fungi* Trichoderma viride* has cellulolytic potential to be used in bioprocesses that aim at obtaining enzymes for later use for bioconversion of cellulose into glucose. This produced enzyme shows viability in comparison with the commercial cellulase enzyme. The synthesis of cellulases by microorganisms from lignocellulosic residues is a process of great interest, representing the search for renewable sources to replace the fossil energetic matrix.

## Figures and Tables

**Table 1:** Enzymatic activity (U/g) of the cellulolytic complex, obtained from the fermented bran, under pH and temperature conditions tested in the Full Factorial Design with three central points.

Experiment	*T* (°C)	pH	Substrate		
			Filter paper	Eucalyptus sawdust	Corncob
1	40	4.4	10.634^d^	8.241^c^	9.200^b^
2	40	5.2	9.629^cd^	10.146^d^	10.785^c^
3	60	4.4	8.421^bc^	7.256^b^	8.271^a^
4	60	5.2	7.762^ab^	6.631^a^	8.992^b^
5	50	4.8	6.649^a^	9.391^d^	9.543^b^
6	50	4.8	6.984^a^	10.118^d^	9.359^b^
7	50	4.8	6.880^a^	10.044^d^	9.086^b^

In the same column, different letters mean statistical difference at 5% significance.

**Table 2:** Enzymatic activity (U/mL) of the commercial cellulase under the conditions of pH and temperature tested in the 2^2^ Full Factorial Design with three central points.

Experiment	*T* (°C)	pH	Substrate		
			Filter paper	Eucalyptus sawdust	Corncob
1	40	4.4	12.807^bc^	3.276^d^	26.87^b^
2	40	5.2	13.563^cd^	3.272^d^	25.308^b^
3	60	4.4	8.805^a^	1.369^b^	10.423^a^
4	60	5.2	11.315^b^	1.181^a^	16.099^a^
5	50	4.8	15.771^e^	3.692^e^	25.456^b^
6	50	4.8	15.419^de^	2.914^c^	26.281^b^
7	50	4.8	15.528^de^	3.121^d^	24.310^b^

In the same column, different letters mean statistical difference at 5% of significance.

**Table 3:** Analysis of variance and effects estimated from variables pH and temperature on the enzymatic activity in the experiments of the Full Factorial Design 2^2^ with three central points.

Substrates	Source of variation	Cellulolytic complex		Commercial enzyme	
		Effect estimated	Level of significance *P* < 0.05	Effect estimated	Level of significance *P* < 0.05
Filter paper	*X*_1_	−2.040	0.322	−1.225	0.673
	*X*_2_	−0.832	0.663	2.745	0.373
	*X*_1_ · *X*_2_	0.173	0.926	2.674	0.384

Eucalyptus sawdust	*X*_1_	−2.250	0.203	−1.972	0.078
	*X*_2_	0.640	0.676	−0.069	0.932
	*X*_1_ · *X*_2_	−1.265	0.429	−0.119	0.883

Corncob	*X*_1_	−1.361	0.005	−12.787	0.059
	*X*_2_	1.153	0.009	2.098	0.665
	*X*_1_ · *X*_2_	−0.432	0.105	3.578	0.474
